# Seasonal variation in the diet of estuarine bivalves

**DOI:** 10.1371/journal.pone.0217003

**Published:** 2019-06-17

**Authors:** Alexa Sarina Jung, Henk W. van der Veer, Marcel T. J. van der Meer, Catharina J. M. Philippart

**Affiliations:** 1 NIOZ Royal Netherlands Institute for Sea Research, Department of Coastal Systems, Utrecht University, AB Den Burg, Texel, The Netherlands; 2 NIOZ Royal Netherlands Institute for Sea Research, Department of Microbiology & Biogeochemistry, Utrecht University, AB Den Burg, Texel, The Netherlands; 3 University of Utrecht, Department of Physical Geography, TC Utrecht, The Netherlands; Universidade de Aveiro, PORTUGAL

## Abstract

Estuarine food webs are generally considered to be supported by marine pelagic and benthic primary producers and by the import of dead organic matter from the open sea. Although estuaries receive considerable amounts of freshwater phytoplankton and organic compounds from adjacent rivers, the potential contribution of these living and dead matter to estuarine food webs is often assumed to be negligible and, therefore, not examined. Based on stable isotope analyses, we report the importance of freshwater suspended particulate organic matter (FW-SPOM) for fuelling estuarine food webs in comparison to estuarine SPOM and microphytobenthos. This previously neglected food source contributed 50–60% (annual average) of food intake of suspension-feeding bivalves such as cockles (*Cerastoderma edule*), mussels (*Mytilus edulis*) and Pacific oysters (*Magallana gigas*) at the Balgzand tidal flats, an estuarine site in the western Wadden Sea (12–32 psu). For these species, this proportion was particularly high in autumn during strong run-off of SPOM-rich freshwater, whilst estuarine SPOM (20%-25%) and microphytobenthos (15%-30%) were relatively important in summer when the freshwater run-off was very low. These findings have implications for our understanding of the trophic interactions within coastal food webs and for freshwater management of estuarine ecosystems.

## Introduction

Coastal ecosystems and their food webs are under the influence of a variety of tidal and seasonally fluctuating factors in environmental conditions (e.g. temperature, salinity and hydrodynamics, [1]). In addition, long-term (interannual) variation in coastal community structures due to loss or gain of biodiversity (e.g. extinction/disappearance of species, invasions) and anthropogenic changes (e.g. input of nutrients, extraction of biomass by fisheries) occur [2–9]. These variations at different time scales will have consequences for the trophic transfer, isotopic niches and predator-prey interactions within the food web. However, to identify long-term changes from higher-order dynamics is a challenge. This is particularly true for marine organisms such as macrozoobenthos for which long-term trend analyses are based upon data that are gathered once a year during a particular season.

Considering coastal marine bivalves, for example, the sampling is often restricted to one (e.g. [10, 11]) or two periods (e.g. [12, 13]) in a year. If the seasonal timing of spatfall varies between years, then recruitment success (spat m^-2^) might be over- or underestimated if sampling is performed at a fixed date [14]. If the diet of a marine coastal bivalve depends on body size (e.g. as found for *Limecola balthic*a by [15]) then, as a consequence, the estimate for the total annual trophic transfer by the local stock will be related to the timing of sampling. If the bivalves are opportunistic in their diet and/or the availability of various food sources varies throughout the season, then determination of main diet based upon observations during one season (e.g. [16]) might be biased. In order to extrapolate seasonal values to annual estimates of trophic transfer, insights into the seasonality of the diet of coastal marine bivalves are required.

Based upon the length of their siphons compared to their burrowing depth, coastal marine bivalves are often divided into suspension feeders (filtering suspended particulate organic matter out of the water column with their relatively short siphons) and facultative deposit-feeders (able to graze from sediment surface using their relatively long siphons). Although bivalves prefer living microalgae (e.g. [17–21]), detritus might still be part of their diet, even if originating from vascular plants [22]. For many bivalve species, main food sources in their direct benthic environment can vary through the year due to sedimentation of pelagic microalgae and organic matter during slack tides and/or calm weather [23, 24], to wind and tide-driven resuspension of benthic matter [18, 24–26], and to seasonal variation in burrowing behaviour of the facultative deposit-feeders [27, 28]. Competition of coexisting intertidal bivalves for food might, therefore, differ throughout the year as the result of external (availability of food sources) and internal (e.g. siphon length, shell size and burrowing depth) variation.

Until a few decades ago, stomach content analysis was the main tool to determine the diet of coastal marine bivalves [18, 29, 30]. However, the approach is very time-consuming and provides diet information of a snapshot in time and requires detailed taxonomic knowledge of prey items [31]. Since the 1970s, the composition of stable nitrogen (^15^N) and carbon (^13^C) isotopes of various potential food items in relation to bivalve body mass is used to estimate the trophic position of an organism as well as the source of its diet, integrated over a longer time period [32]. Because freshwater algae (-36 to -30 ‰) have lower δ^13^C values than marine algae (-24 to -20 ‰) and marine microphytobenthos (-20 to -16 ‰) [20, 33], food source of coastal marine bivalves can often be determined by analysing their stable isotope signature [20, 21, 34–36].

**Fig 1:**
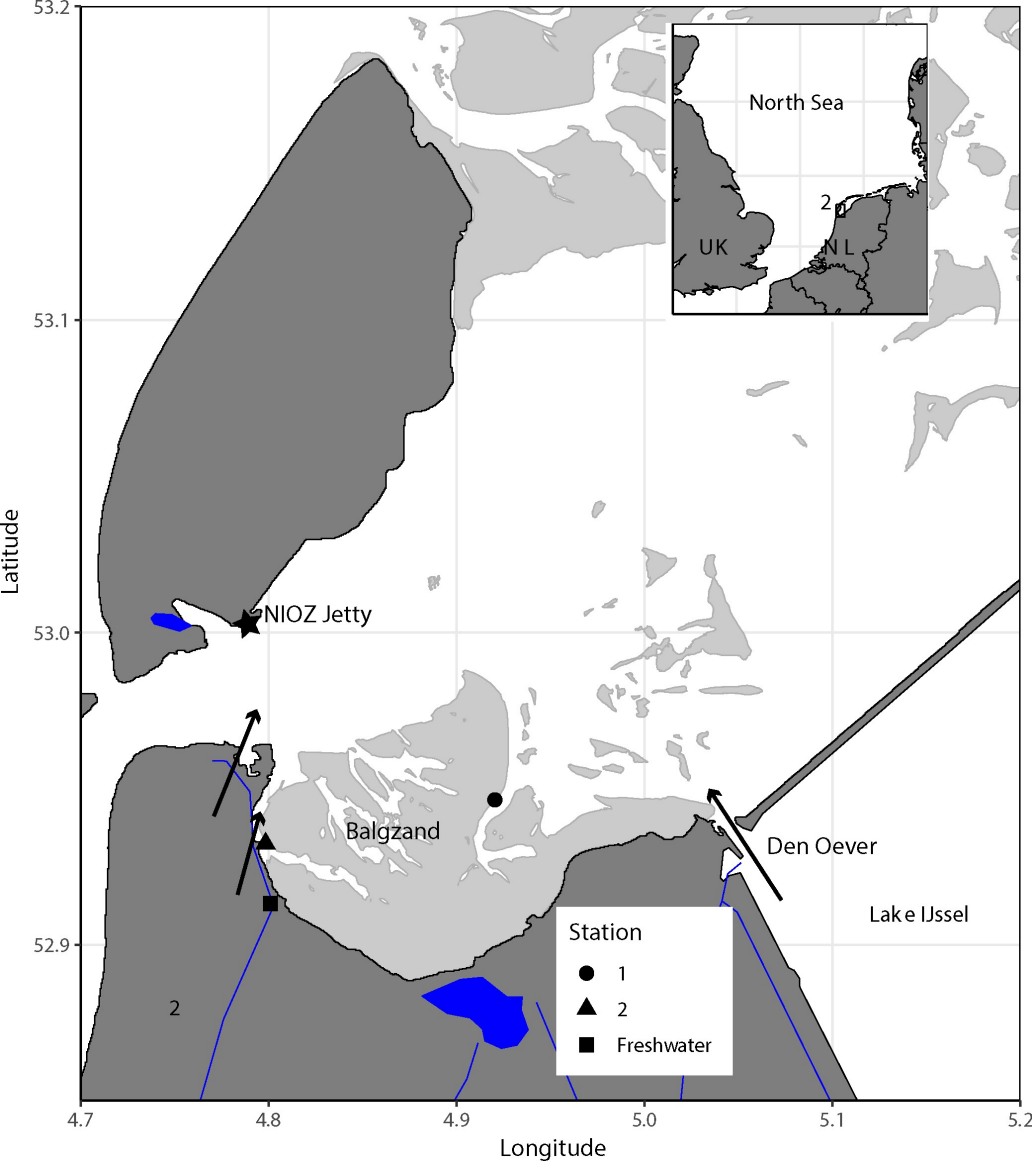
Map of the study area. The two sampling stations on the Balgzand tidal flats are indicated, as well as the freshwater station and the NIOZ jetty (star). The arrows depict the locations (sluice in Den Oever, the Balgzand channel and the harbour of Den Helder) where freshwater is discharged into the western Wadden Sea.

In this study, we expand on the diet studies from the past [18] and explore the seasonality in the diet of coastal marine bivalves living on the Balgzand tidal flats in the western Wadden Sea using stable isotope analyses (Fig 1). Because this area is under the influence of freshwater outputs of Lake IJssel [37–39], we have included freshwater SPOM samples to explore the proportion of this potential food source to estuarine SPOM and benthic organic matter [40–43]. Seasonal variation in relationships between diet and body size are investigated for all sampled bivalve species, as are trophic niches within and between bivalve species throughout the year. We discuss our results on seasonality in diet with respect to potential food availability, to previous statements on main diet and trophic transfer in this area and to the consequences of using seasonal samples to describe long-term variations in trophic interactions in general.

## Material and methods

### Study area

All sampling took place at the Balgzand, a large (50 km^2^) isolated tidal flat system bordering the Marsdiep tidal inlet in the western part of the Wadden Sea (Fig 1). The area is surrounded by dikes to the south and west and by tidal channels ranging in depth from 5 to 20 m (relative to mean sea level). Median grain size and silt content of the sediment are about 150 μm and 5%, respectively, with a gradient from coarse sand at more exposed flats in the north to fine sands and mud at the more sheltered areas in the south [10]. On average, the tidal flats are drained for 2 to 4 hours of the 13-hour tidal cycle. At high tide, most of the area is covered by 0.7 to 1 m of water, the actual depth depending on the lunar phase and weather conditions.

The Balgzand tidal flat area has been studied by the Royal Netherlands Institute for Sea Research (NIOZ) since the early 1970s for macrozoobenthos [44–46], crustaceans [47–49], and fish [47, 50, 51]. These long-term studies revealed strong year-to-year variation and multi-annual trends, including the impacts of long-term variation in nutrient supply [52–55], the invasion and establishment of Pacific oysters [56] and the shift from juvenile flatfish to shrimps as main predators of small macrozoobenthic prey [47]. Since 2008, the Balgzand tidal flats are also covered by the SIBES (Synoptic Intertidal BEnthic Surveys of the Wadden Sea) surveys, during which intertidal macroozoobenthos is sampled in summer (July/Aug) in a grid of 500 m x 500 m [57]. In addition to the long-term field observations, this area was subject to a suite of field experiments and other in-depth studies, including studies on the diet of bivalves based upon stomach analyses [18, 29].

### Sampling

Field work was conducted under the general permit (permit number 01028628) for the Costal Systems department of the NIOZ for 2012–2015. The permission was granted by the department “Stéd en Plattelân” of the “provinsje fryslân” at 7 December 2012 (for 3 years). At the Balgzand tidal flats, samples were collected around high (temperature, salinity, suspended particulate organic matter, SPOM; Table 1) and low water (macrozoobenthos, microphytobenthos; Table 2) at two stations (Fig 1) during four different periods in time (March, June, September and December of 2014), either by foot or from board of the *R*.*V*. *Stern* and rubber dinghies (Table 1). In addition, freshwater SPOM samples were collected (one sample per period) one day after the estuarine SPOM samples in a small channel that is supplying freshwater to the Balgzand tidal flats (52.913° N, 4.801° E; Fig 1).

**Table 1:** Sampling dates, local sampling times, mean temperature (°C), mean salinity (PSU) and the number of estuarine SPOM samples collected at high tide at two estuarine stations (Fig 1) at Balgzand tidal flats in 2014. Freshwater SPOM samples were taken one day after the estuarine SPOM samples.

Station	Date	Time span	Mean Temperature	SD	Mean Salinity	SD
1	13.03.2014	09:00–09:40	7.65	0.15	16.55	0.15
2	12.03.2014	10:20–10:50	8.55	0.05	19.95	2.26
1	27.06.2014	11:00–12:15	18.40	0.24	23.72	0.97
2	23.06.2014	13:00–14:10	19.53	0.12	11.77	2.18
1	23.09.2014	09:10–09:40	14.64	0.10	21.67	0.1
2	18.09.2014	12:20–13:20	19.73	0.09	22.85	1.92
1	15.12.2014	12:50–13:20	6.33	0.12	31.14	0.14
2	16.12.2014	12:40–13:30	7.27	0.17	31.55	0.13

**Table 2:** Sampling dates, number of bivalve species and total number of bivalves sampled at low tide at two estuarine stations (Fig 1) at Balgzand tidal flats in 2014.

Station	Date	N bivalve species	N bivalves sampled
1	13.03.2014	5	74
2	12.03.2014	6	46
1	27.06.2014	6	25
2	23.06.2014	7	38
1	23.09.2014	6	21
2	18.09.2014	7	47
1	15.12.2014	3	7
2	16.12.2014	5	17

Estuarine and freshwater SPOM samples were collected from surface water samples with a bucket. For the estuarine samples, water temperature and salinity were measured with handheld automated devices (Delta Ohm HD2105.1 and Delta Ohm HD2105.2). This information was not gathered for the freshwater SPOM samples. Samples were sieved through a 200 μm mesh to exclude larger zooplankton from the sample and stored in an ice chest. In the lab samples were filtered onto pre-combusted 25 mm GF/F filters using a 25 mm filter cartridge mounted on a 60 ml syringe. Between 80 and 250 ml of water was filtered, samples were stored at -20°C until further processing.

Samples for microphytobenthos (MPB) were sampled by collecting the top layer of sediment from visible diatom mats into plastic bottles that were brought back to the research facility cooled. Microphytobenthic diatoms were extracted in the laboratory using the method of Riera and Richard (20), slightly modified by Herlory, Richard [58]. The sediment was spread on a tray, covered by three layers of nylon mesh (2 x 100 μm, 1 x 50 μm) that was kept moist by repeatedly spraying filtered seawater on top. The samples were then left in a temperature regulated room reflecting outside temperature (depending on season 6–20°C) overnight and the following day the algae were washed into a beaker with filtered seawater, concentrated by centrifugation (10 min at 1000x g) and stored at -20°C [59].

Sampling of bivalves was conducted around low water by foot on the dry tidal flats. An area of approximately 20 m^2^ was searched for living animals by collecting organisms on the surface (e.g. the epibenthic *Magallana gigas*, formerly known as *Crassostrea gigas*), by looking for siphon holes or other structures (e.g. the infaunal *Mya arenaria*, of which large specimens burrow up to a depth of 25 cm [60]), or by randomly sieving the upper 5 cm of sediment over a 1 mm^2^ sieve. The collected organisms were transported in separate plastic containers and taken to the lab, where they were washed and stored in filtered seawater for 24–48 h to get rid of stomach and gut contents. They were then measured (nearest mm), shells were removed from bivalves larger than 0.5 cm and organisms were stored separately in glass vials at -20°C until stable isotope analyses.

### Seasonality in primary sources of microalgae

Seasonality in biomass of potential sources of living pelagic and benthic microalgae (marine pelagic microalgae, freshwater microalgae, microphytobenthos) was based upon relative seasonal variations (compared to the maximum value observed) in chlorophyll-*a* concentrations of phytoplankton in the Marsdiep tidal inlet and in Lake IJssel, respectively, and NDVI (Normalized Density Vegetation Index) values for the intertidal area of the Dutch Wadden Sea (Table 1).

Seasonal variation in estuarine phytoplankton biomass in 2014 (mg Chl *a* m^-3^) was derived from the long-term field observations at the NIOZ jetty (Fig 1; see [61] for materials and methods). Since sampling was performed at the tidal inlet at high water, these values are expected to have a relatively high component of materials of marine origin.

Seasonal variation in supply of freshwater algal biomass was calculated as the discharge of freshwater (m^3^ month^-1^; live.waterbase.nl) from Den Oever, a sluice complex approximately 25 km east of the Balgzand tidal flats (Fig 1), multiplied by average monthly concentrations of chlorophyll-a (mg Chl *a* m^-3^; live.waterbase.nl) at the sampling station Vrouwezand (52.810350° N, 5.393138° E) in the southern part of Lake IJssel (Fig 1, station not shown) in 2014.

Because microphytobenthos biomass (mg Chl *a* m^-2^) was not available for 2014, we based the seasonality of benthic microalgae on NDVI values as derived for the period 2002–2004 from satellite images by van der Wal et al. [62].

The possible fate of freshwater SPOM in seawater (deterioration) was explored by plotting the variations in δ^15^N and δ^13^C values of estuarine suspended particulate organic matter (SPOM) as a function of local salinity, including the values of the freshwater SPOM as reference levels.

### Stable isotope analysis (SIA)

Prior to the Stable Isotope Analysis (SIA), all samples were freeze dried for 48 hours at -60°C to remove water, homogenized and decalcified if necessary. Samples were weighted and folded into tin cups for analysis except for samples that needed to be decalcified. Those samples were decalcified in silver cups with 1 M HCl to remove inorganic carbonate and dried for another 24 h at 60°C, folded and analysed. Nitrogen and carbon isotope ratios for all samples were determined with a Thermo Scientific Delta V Advantage Isotope Ratio Mass Spectrometer linked with a Flash 2000 Organic Element Analyzer at the NIOZ. Isotope ratios (R) are presented in the δ notation in ‰ relative to an internationally defined reference.

$$\delta X=\left(R_{sample}/R_{reference}-1\right)\;*\;1000$$

with R being the ratio between the heavy and light isotopes (^15^N:^14^N or ^13^C:^12^C). The reference for δ^15^N is atmospheric nitrogen and for δ^13^C is Vienna Peedee-Belemnite (VPDB). For each analytical run monitoring gas, both N_2_ and CO_2_, with a predetermined isotopic composition was used to determine the δ values for all standards and samples. Three standards with known isotopic composition were included at the beginning, after every twelve samples and at the end of each analytical sequence to monitor performance of the machine and to correct for the offset between the measured and actual isotope ratio. One standard, Acetanilide, was used to correct the measured values and the other two standards, Urea and Casein, to check the correction. Analytical reproducibility was 0.3 ‰ for δ^15^N and 0.1 ‰ for δ ^13^C throughout every sequence. Before the standards each sequence starts with multiple blanks, empty tin cups, to remove air if present and to determine a potential blank contribution to the analysis. Blanks are typically too low to be of any importance.

### Data analysis

To establish that the isotope values of the three sources were different they were tested using an ANOVA, when significant this was followed by a pairwise Tukey Honest Significant Differences test corrected for multiple comparisons.

To directly compare the stable isotope signal of the bivalves with their potential food sources, we assumed a trophic fractionation factor of 3.78 ‰ for δ^15^N and 2 ‰ for δ^13^C per trophic level [63].

To determine the relative contribution of the various food sources to the diet of the bivalves, an isotopic mixing model was applied for each season to the bivalve data available by using the R package simmr (Version 0.3, [64, 65]). This package is designed to solve mixing equations for stable isotopic data within a Bayesian framework, and includes the possibility of *a posteriori* testing of correlations between probabilities of different sources [66].

The relationship between the shell length of a bivalve and the δ^13^C and δ^15^N values was tested for all samples of one species by fitting a linear model through the data according to:

$$\delta^{13}C={ß}_{1}•Shell\;length\left(mm\right)+factor\left(Season\right)$$

$$\delta^{15}N={ß}_{2}•Shell\;length\left(mm\right)+factor\left(Season\right)$$

where ß_1_ represents the slope of change in δ^13^C per mm, and ß_2_ the slope of change of δ^15^N per mm.

The size of the isotopic niche of each bivalve species was analysed for each season separately with the R package SIBER (Version 2.1.3 [65, 67]). Only bivalve species with more than 4 samples were included in the analysis.

Trophic niche of all bivalve species was investigated by calculating the Standard Ellipse Area corrected for small sample sizes (SEA_*c*_) from δ^15^N and δ^13^C measurements of individuals. This is a bi-variate measure of variation similar to the standard deviation [67].

Variation in seasonal diet overlap within species and between species was investigated by calculating the percentage of overlap of the area of the SEA_*c*_ ellipses of one species in the course of the year and all species for each season separately, respectively.

## Results

### Seasonality in primary sources of microalgae

**Fig 2:**
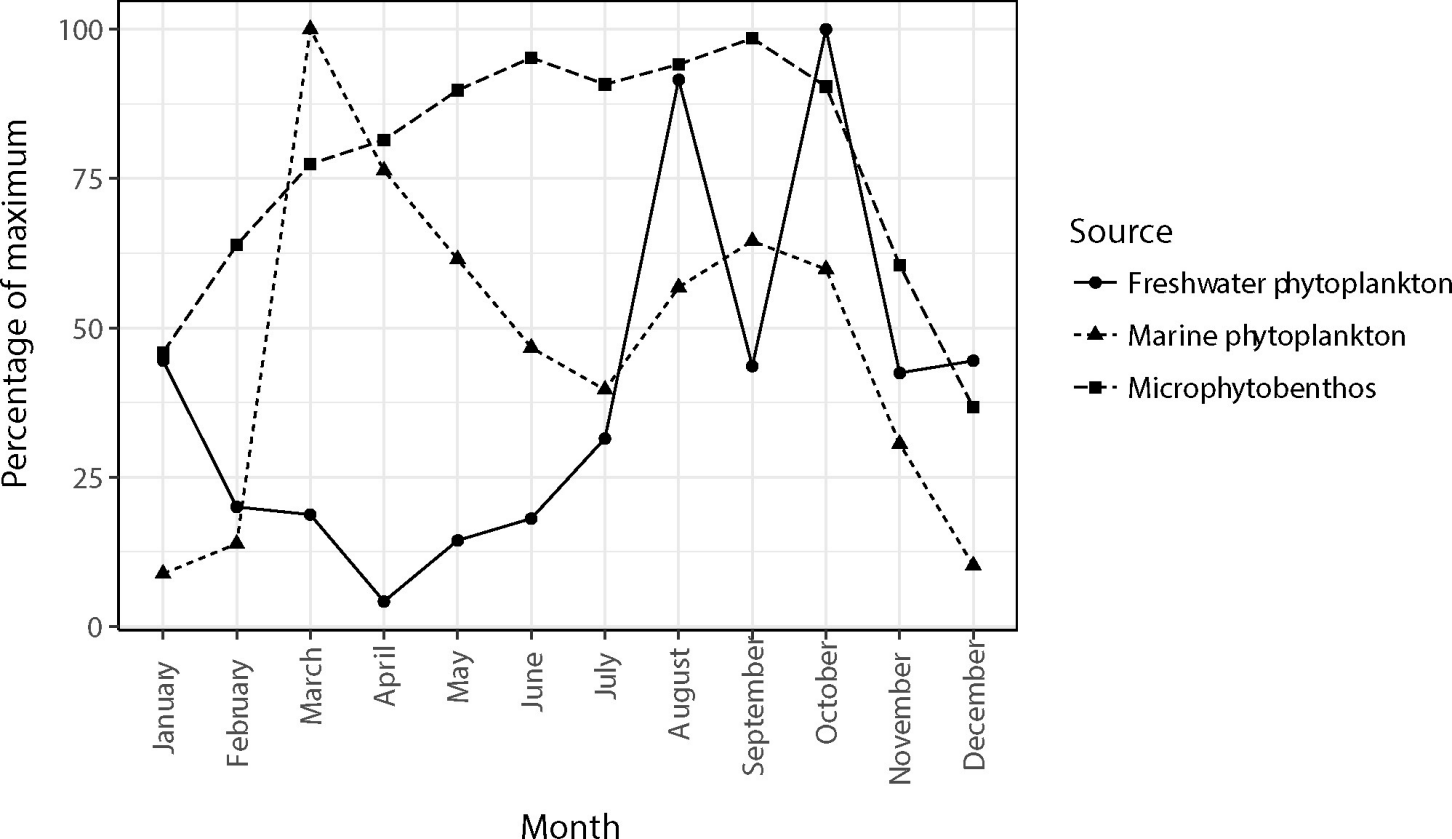
Monthly variations in living microalgae as food for the Balgzand bivalve community. Values are relative to their respective maximum (set as 100%) of chlorophyll-*a* concentrations (mg Chl *a* m^-3^) in the Marsdiep tidal inlet in 2014 (marine phytoplankton), NDVI of the tidal flats of the Dutch Wadden Sea between 2002 and 2004 (microphytobenthos) and input of chlorophyll-a (mg Chl *a* month^-1^) from Lake IJssel via the discharge sluices at Den Oever (freshwater phytoplankton). See text for data sources.

In 2014, the chlorophyll-a concentrations within the Marsdiep tidal inlet peaked in March (14.1 μg L^-1^, Fig 2) after which a steady decline occurred until July (40% of maximum, Fig 2). A small autumn bloom could be detected with a peak in September (9.1 μg L^-1^, 64% of maximum, Fig 2), the minimum concentration was found in January and December (Fig 2).

In 2014, peak chlorophyll-a discharge from Lake IJssel via the sluice at Den Oever was found in autumn (August 19.6 μg Chl *a* s^-1^ and October 21.4 μg Chl *a* s^-1^) and lowest values were observed in April (0.9 μg Chl *a* s^-1^, 4% of maximum, Fig 2).

In the period 2002–2004, NDVI values of microphytobenthos at the Balgzand flats were highest during the summer/autumn period (June-October) with values up to 0.14 NDIV (Fig 2). Comparable to the marine phytoplankton biomass, the lowest NDVI values were found in January (46%) and December (36%).

The three primary sources for local concentrations of living microalgae at Balgzand show distinctively different seasonal patterns with respective peaks in spring (marine phytoplankton), summer/autumn (microphytobenthos) and autumn (freshwater phytoplankton).

**Fig 3:**
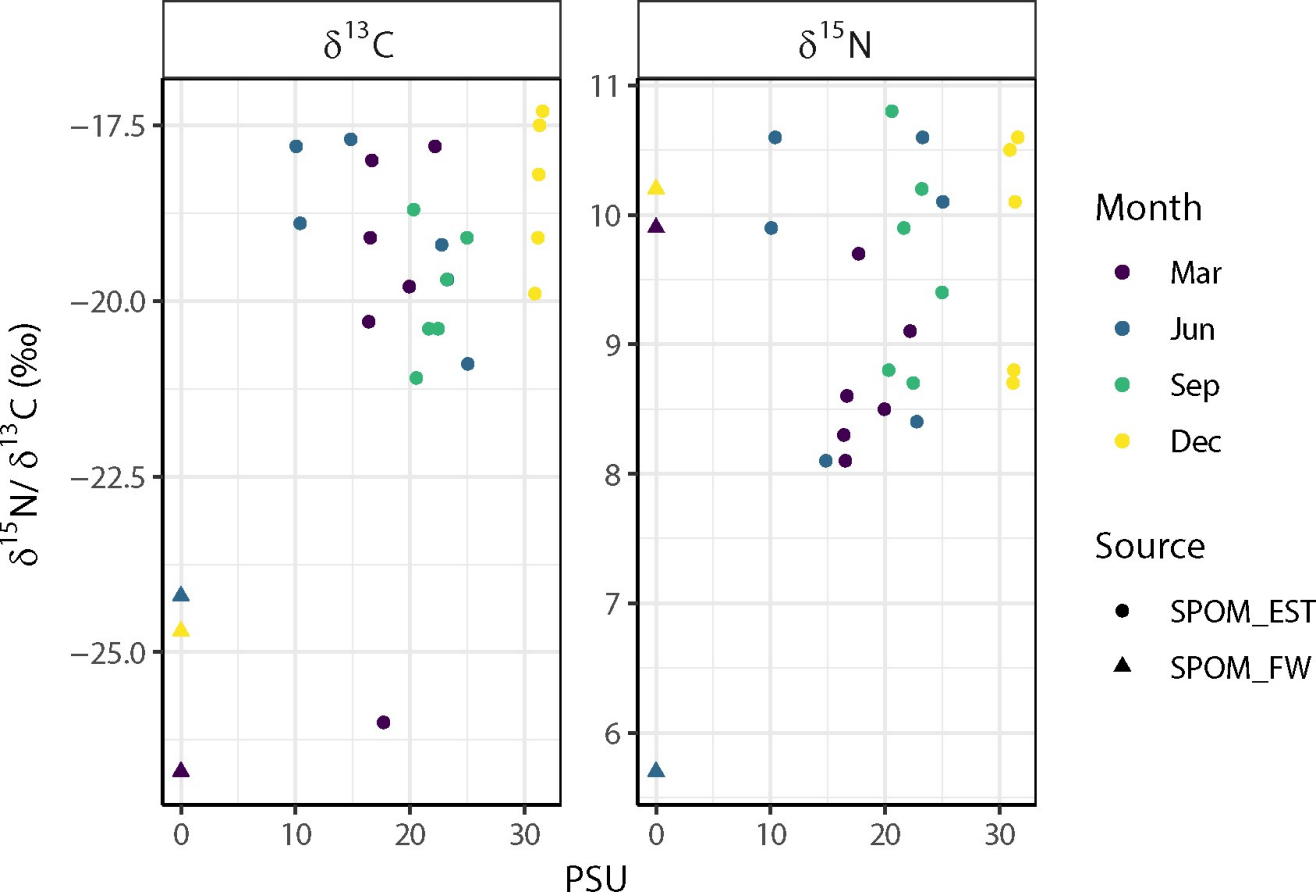
**Variations in δ**^**13**^**C (left panel) and δ**^**15**^**N (right panel) and values of estuarine suspended particulate organic matter (SPOM_EST) as a function of local salinity.** The triangles denote the stable isotope values of the freshwater SPOM (SPOM_FW) samples.

There were no significant linear relationships between stable isotope signals of the estuarine SPOM and salinity (Fig 3). On average, however, the values of the δ^13^C values of the freshwater SPOM were lower than the δ^13^C values of the estuarine SPOM (Fig 3). The means of δ^13^C of all three sources had significantly (p < 0.05) different values, whereas the means of δ^15^N were not significantly different between the sources.

### Seasonality in stable isotope signatures of SPOM and microphytobenthos

**Fig 4:**
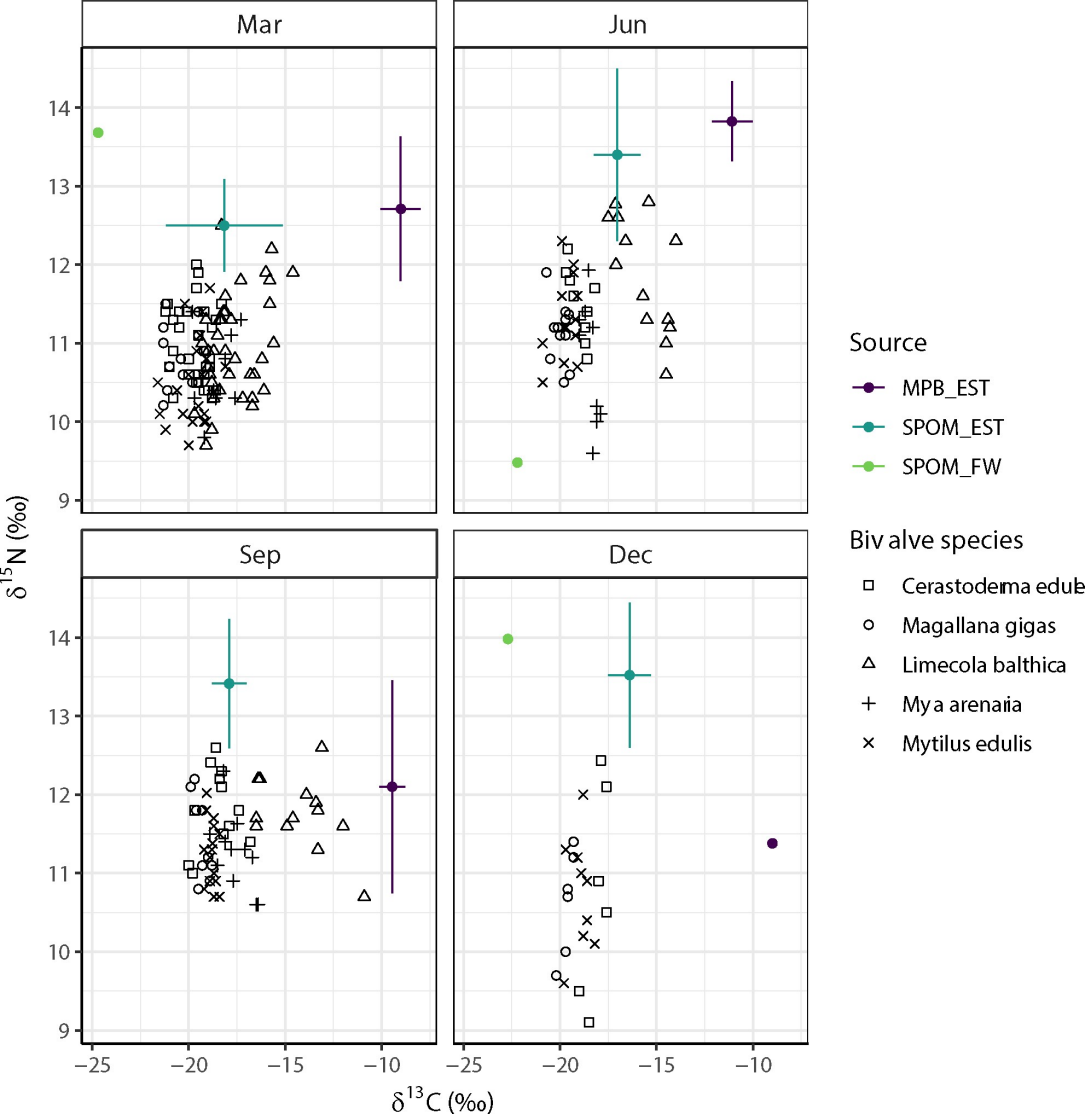
Tracer plots of the estuarine bivalve species as sampled in March, June, September and December 2014 at the Balgzand tidal flats in relationship to local pelagic (SPOM_EST) and benthic (MPB_EST) samples and to pelagic samples taken in an adjacent freshwater channel (SPOM_FW). Note that the isotope signals of the SPOM (estuarine and freshwater) and microphytobenthos samples are already corrected for trophic enrichment by one trophic level (+3.78‰ δ^15^N and +2‰ δ^13^C; [63]).

Seasonal variation in the average stable isotope signals of the SPOM (estuarine, freshwater) and microphytobenthos samples was most pronounced in the nitrogen isotopic signatures of the freshwater SPOM. The δ^15^N value in June (5.7‰) was almost 5 ‰ lower than those in March (9.9‰) and December (10.2 ‰) (Fig 4). Unfortunately no information is available for September. The average of March, June and December FW_SPOM was used for further analyses if needed. Average δ^15^N values of the estuarine SPOM samples varied between a relatively low 8.7‰ in March to more than 9.6‰ for all other seasons, whilst those of microphytobenthos ranged between 7.6‰ in December to more than 10‰ in June (Fig 4). Variation in the average δ^13^C values was around 2‰ in all three types of samples (Fig 4).

### Seasonality in stable isotope signatures of estuarine bivalves

**Fig 5:**
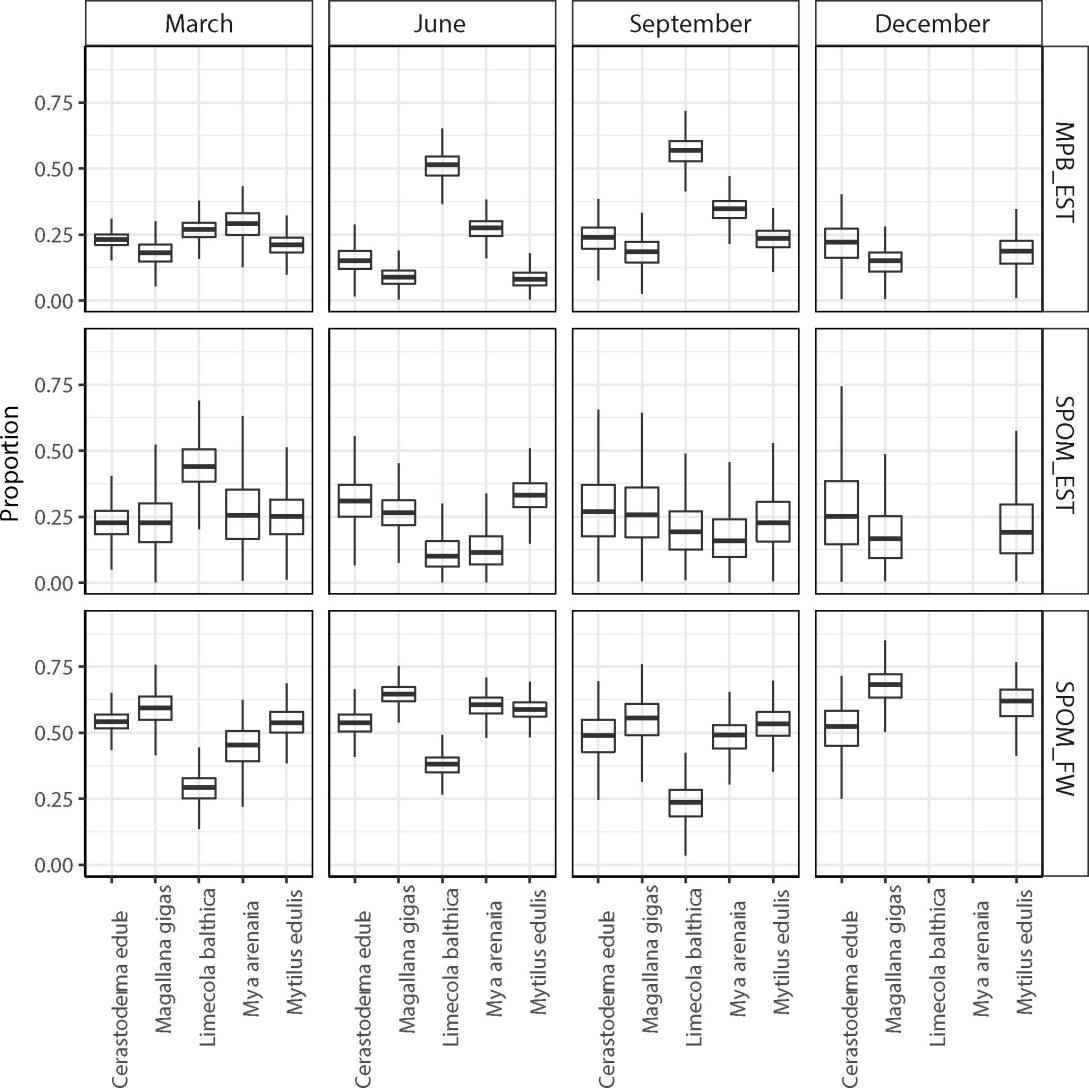
Relative contribution of different food sources to the diet of estuarine bivalves at the Balgzand tidal flats in March, June, September and December 2014.

Stable isotope signatures of the estuarine bivalves were more or less centred around the signatures (corrected for trophic enrichment) of the estuarine SPOM samples, in particular in March and June (Fig 4). The stable isotope mixing model results suggest that for *Limecola balthica*, estuarine SPOM is the most probable food source in March (>0.45; Fig 5) however, the diet of this facultative deposit feeder switched to benthic microalgae in June and September (> 0.50; Fig 4). For all other bivalve species sampled during all periods of the year (*Cerastoderma edule*, *Magallana gigas*, *Mya arenaria*, *Mytilus edulis*), the results indicated a high probability of feeding mainly on freshwater SPOM (approximately 0.5) up to more than 0.7 for *M*. *gigas* in December (Fig 5).

Season-specific matrix plots of food sources showed, however, mostly high (r > 0.65) negative (between microphytobenthos and estuarine SPOM, between estuarine and freshwater SPOM) and positive (between microphytobenthos and freshwater SPOM) correlations (S1 Fig). For several occasions the positive correlation between microphytobenthos and freshwater SPOM was relatively low (< 0.3), for *L*. *balthica* in June and September (S1 Fig).

### Correlation of δ^13^C and δ^15^N with shell length of estuarine bivalves

**Fig 6:**
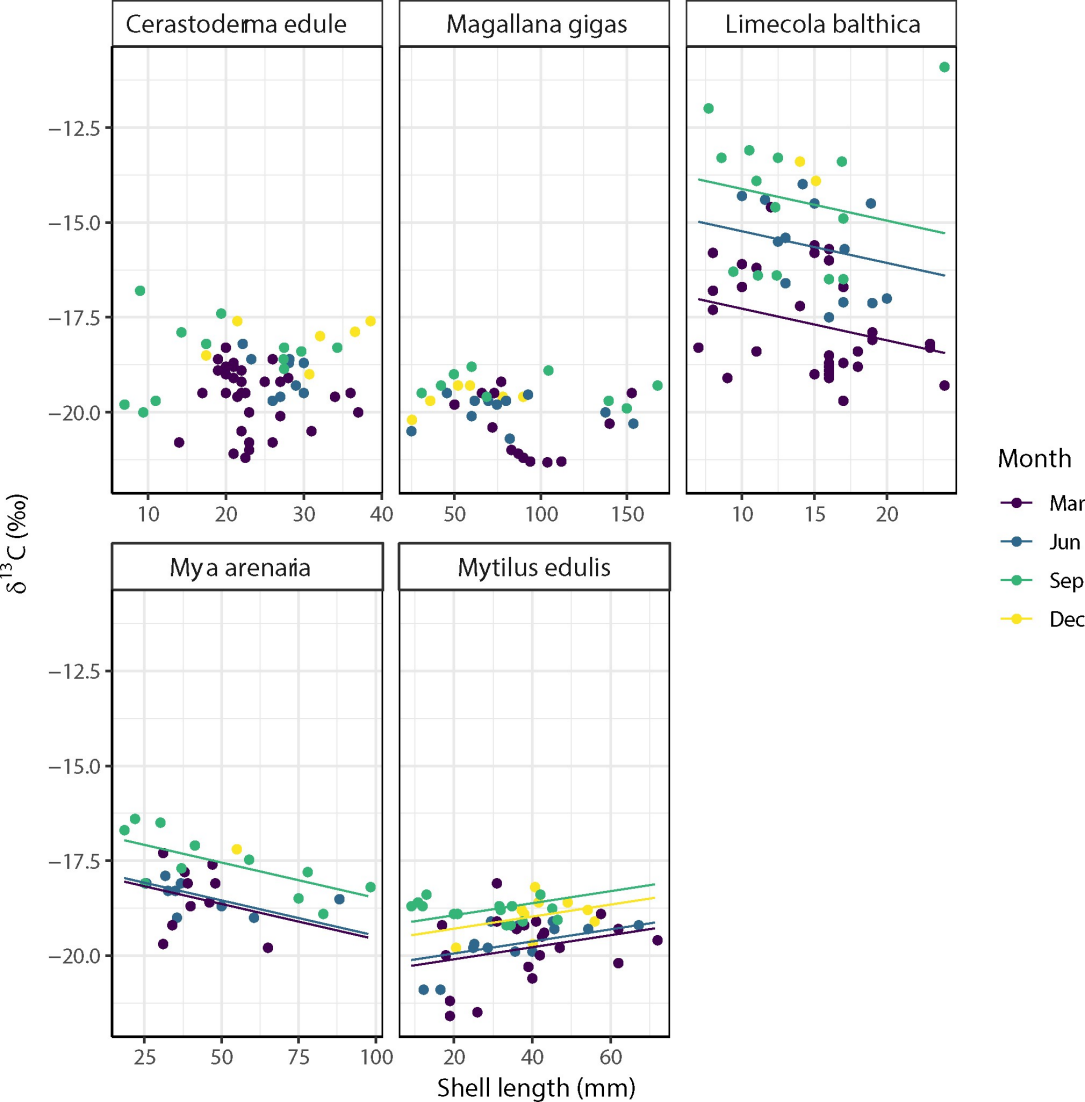
Relationships between the δ^13^C signals and shell length estuarine bivalves at the Balgzand tidal flats in March, June, September and December 2014. Linear relationships with significant (p < 0.05) slopes are plotted as solid lines (see S1 Table in the supporting information for significance of these relationships). For *M*. *edulis*, the δ^13^C signal significantly increased with shell length (+0.02 δ^13^C mm^-1^) with the intercept of this relationship being higher in September and December as in March (Fig 6, S1 Table). The slopes of these relationships were not significant for the other three estuarine bivalve species examined (Fig 6, S1 Table).

For *L*. *balthica* and *M*. *arenaria*, the δ^13^C signal significantly decreased with shell length, with respective slopes of -0.08 δ^13^C mm^-1^ and -0.02 δ^13^C mm^-1^ (Fig 6, S1 Table). For the Baltic tellin *L*. *balthica*, the intercept of this relationship was higher in June and September than in March (Fig 6, S1 Table). For the soft-shell clam *M*. *arenaria*, the intercept of this relationship was higher in September than in March and June (Fig 6, S1 Table).

**Fig 7:**
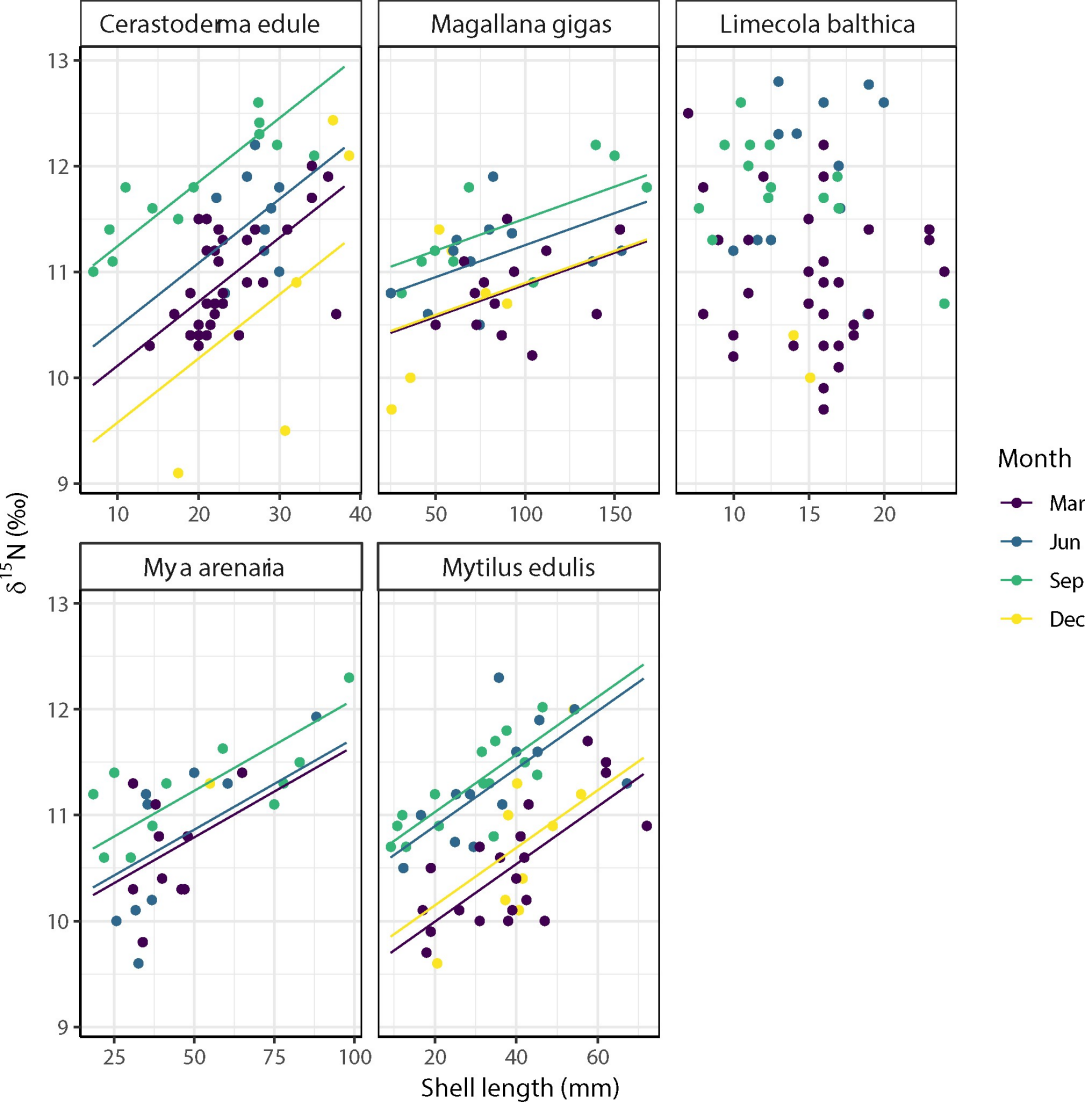
Relationships between the δ^15^N signals and shell length of estuarine bivalves at the Balgzand tidal flats in March, June, September and December 2014. Linear relationships with significant (p < 0.05) slopes are plotted as solid lines (see S2 Table in the supporting information for significance of these relationships).

For four bivalve species (*C*. *edule*, *M*. *gigas*, *M*. *arenaria* and *M*. *edulis*), the δ^15^N signal significantly increased with shell length with respective slopes of +0.06, +0.01, +0.02 and +0.03 δ^15^N mm^-1^ (Fig 7, S2 Table). For the edible cockle *C*. *edule*, the intercept of this relationship was higher in June and September and lower in December compared to March (Fig 7, S2 Table). For the Pacific oyster *M*. *gigas*, and the blue mussel *M*. *edulis*, the intercept of this relationship was higher in June and September and similar in December as in March (Fig 7, S2 Table). For the soft-shell clam *M*. *arenaria*, the intercept was higher in September compared to March and June (Fig 7, S2 Table). The slope of these relationship was not significant for *L*. *balthica* (Fig 7, S2 Table).

### Seasonality in trophic niche overlap

**Fig 8:**
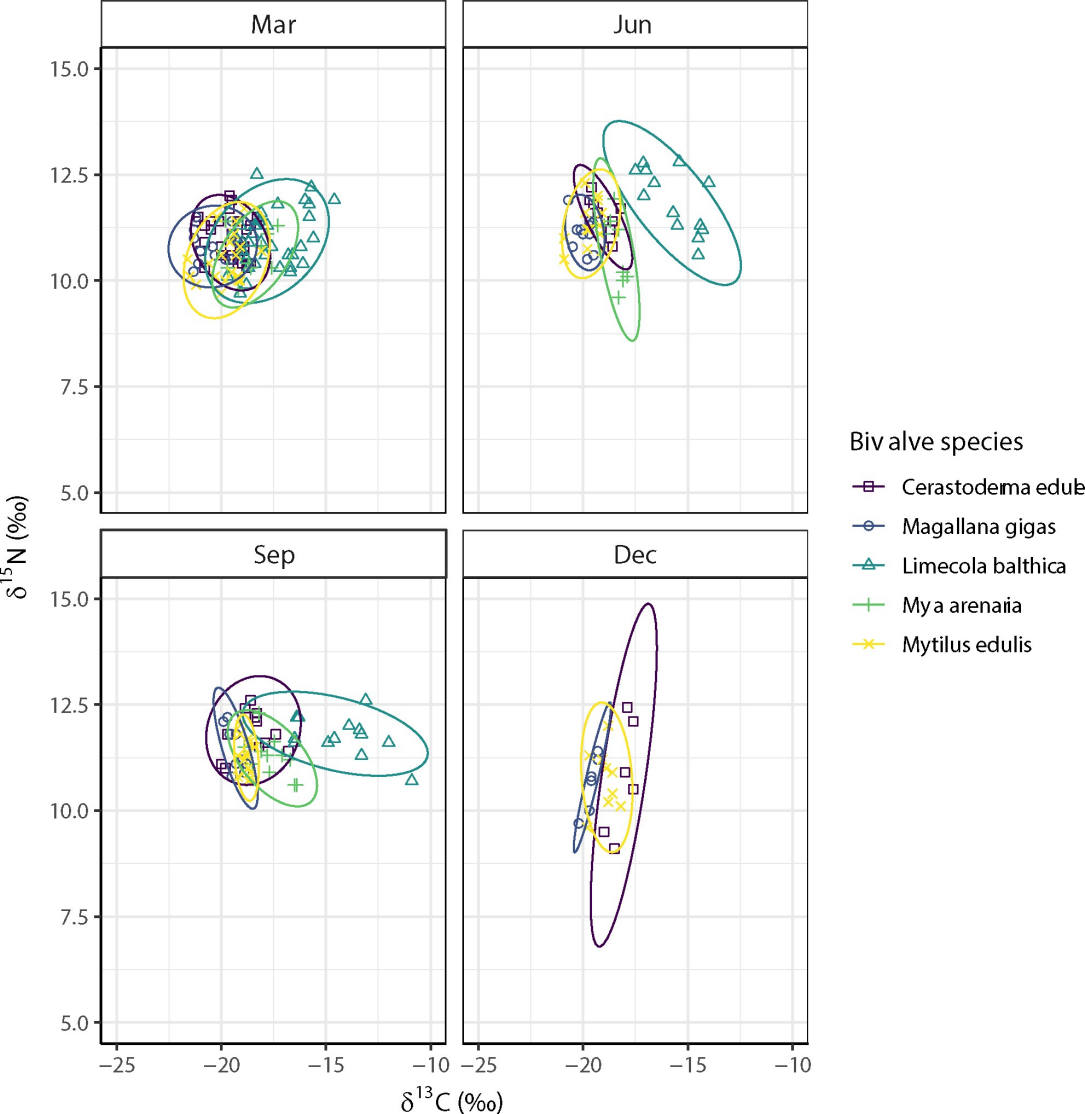
Seasonal variation in isotopic niche overlap between estuarine bivalve species at the Balgzand tidal flats in March, June, September and December 2014.

On average, the SEA_c_ (Standard Ellipse Area corrected for small sample sizes) was highest for the Baltic tellin (*L*. *balthica*) and relatively low for the Pacific oyster (*M*. *gigas*), implying that the isotopic niche of the Baltic tellin was much wider (more than three times) throughout the year than that of the Pacific oyster (Fig 8, Table 3). For *C*. *edule*, the maximum isotopic niche (SEA_c_) in December hardly overlapped (< 40%) the niches in March, June and September, whilst the smallest isotopic niche in June fell mostly (≥ 75%) within those of March and September (Fig 8, Table 3). For *L*. *balthica*, the relatively large isotopic niches (SEA_c_) in the different months only partly overlapped each other, i.e. an overlap between March and June of less than 40% and an overlap between March and September of less than 35%, with June having the largest overlap (>60%) with September (Fig 8, Table 3). For *M*. *gigas*, the relatively large isotopic niche (SEA_c_) in March comprised most of the niches in June (90%), September (58%) and December (60%), but overlap between the isotopic niches of these latter three months was relatively small, i.e. between 17% and 45% (Fig 8, Table 3). For *M*. *arenaria*, the largest isotopic niches in March partly overlapped the ones in June (< 70%) and September (< 65%), the overlap between June and September was less than 50% (Fig 8, Table 3). Also for *M*. *edulis*, the largest isotopic niche in March partly overlapped that of the other months (> 70%), whilst the smallest isotopic niche in September fell largely (> 80%) within those of March, June and December (Fig 8, Table 3).

**Table 3:** Standard Ellipse Area corrected for small sample sizes (SEA_c_, as δ-units^2^) and percent overlap of the SEA_c_ within estuarine bivalve species (%) as sampled in March, June, September and December 2014 at the Balgzand tidal flats.

Species name	Month	SEAc	March	June	September	December
*Cerastoderma edule*	March	7.55		39	56	33
*Cerastoderma edule*	June	3.94	75		94	46
*Cerastoderma edule*	September	10.04	42	37		47
*Cerastoderma edule*	December	11.92	21	15	39	
*Limecola balthica*	March	15.32		37	34	
*Limecola balthica*	June	14.61	39		66	
*Limecola balthica*	September	15.24	34	63		
*Magallana gigas*	March	6.69		41	26	17
*Magallana gigas*	June	3.06	90		39	28
*Magallana gigas*	September	3.05	58	40		25
*Magallana gigas*	December	1.92	60	45	39	
*Mya arenaria*	March	9.11		39	47	
*Mya arenaria*	June	5.13	69		49	
*Mya arenaria*	September	6.79	63	37		
*Mytilus edulis*	March	9.76		47	19	60
*Mytilus edulis*	June	6.37	73		26	66
*Mytilus edulis*	September	2.08	88	81		100
*Mytilus edulis*	December	8.21	71	51	25	

### Seasonality in trophic niche overlap

**Fig 9:**
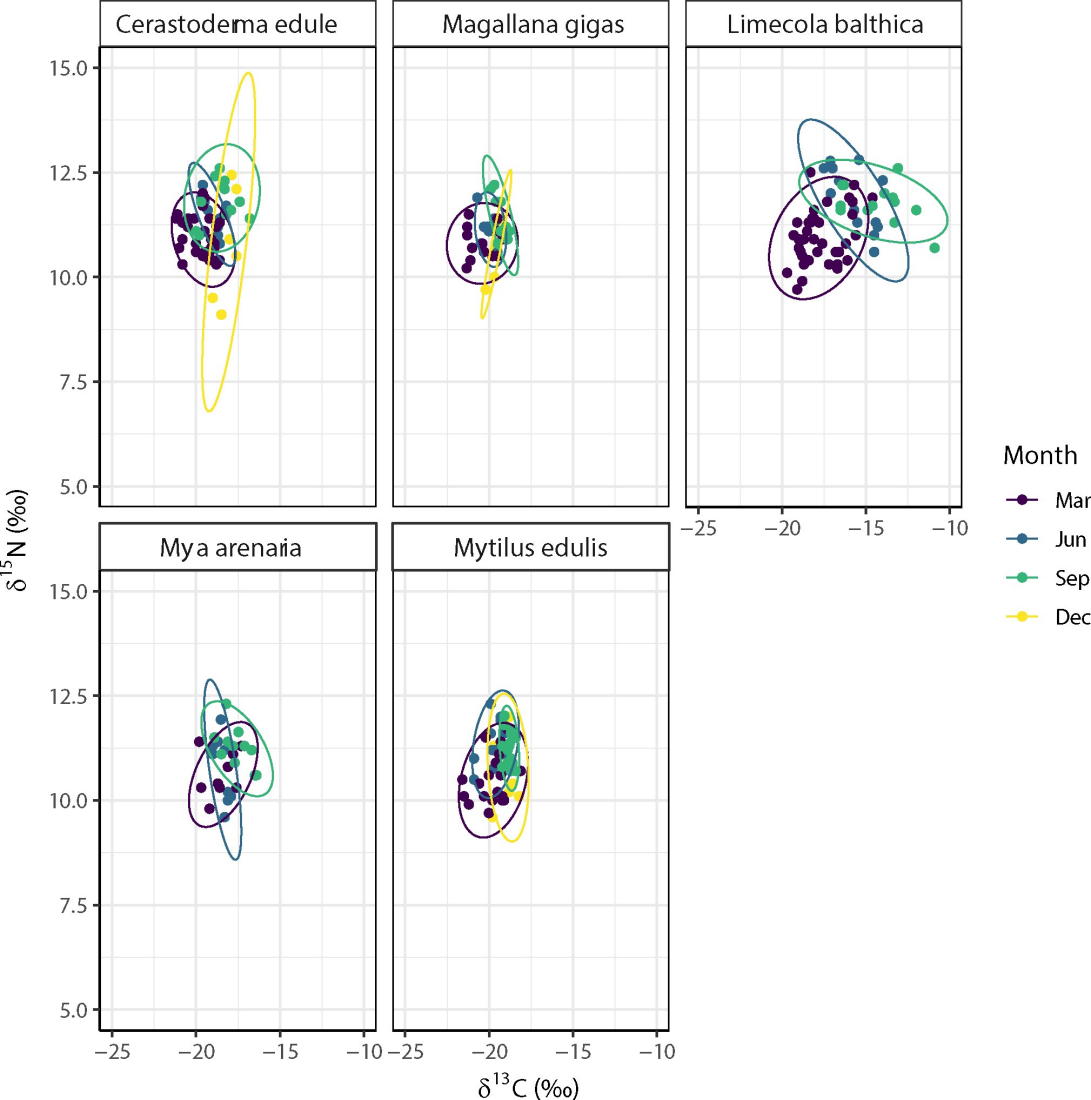
Seasonal variation in isotopic niche overlap within estuarine bivalve species at the Balgzand tidal flats in March, June, September and December 2014.

In March, the relatively large isotopic niche of *L*. *balthica* comprised only part of the niches of the others ranging from 59% for *M*. *arenaria* to 24% for *M*. *gigas* (Fig 9, Table 4).

**Table 4:** Standard Ellipse Area corrected for small sample sizes (SEA_c_, as δ-units^2^) and percent overlap of the SEA_c_ between estuarine bivalve species (%) as sampled in March, June, September and December 2014 at the Balgzand tidal flats.

		SEAc	*Cerastoderma edule*	*Limecola balthica*	*Magallana gigas*	*Mya arenaria*	*Mytilus edulis*
March	*Cerastoderma edule*	7.55		73	67	66	87
	*Limecola balthica*	15.32	36		24	59	42
	*Magallana gigas*	6.69	76	54		48	81
	*Mya arenaria*	9.11	54	99	35		65
	*Mytilus edulis*	9.76	67	66	56	61	
June	*Cerastoderma edule*	3.94		0	24	67	74
	*Limecola balthica*	14.61	0		0	0	1
	*Magallana gigas*	3.06	31	0		11	99
	*Mya arenaria*	5.13	51	1	7		43
	*Mytilus edulis*	6.37	46	2	48	35	
September	*Cerastoderma edule*	10.04		38	26	44	19
	*Limecola balthica*	15.24	25		0	15	1
	*Magallana gigas*	3.05	87	0		37	47
	*Mya arenaria*	6.79	66	33	17		22
	*Mytilus edulis*	2.08	91	9	68	73	
December	*Cerastoderma edule*	11.92		NA	0	NA	39
	*Magallana gigas*	1.92	0				82
	*Mytilus edulis*	8.21	56	NA	19	NA	

In June, the sizes and overlaps in isotopic niches appeared to be smaller than in March, with the smallest overlap being between that of *L*. *balthica* with all other estuarine bivalves, e.g. 0% for *C*. *edule*, *M*. *gigas* and *M*. *arenaria*, 1% for *M*. *edulis* and 8% for *S*. *plana* (Fig 9, Table 4).

In September, the overlap of the relatively large isotopic niche of *L*. *balthica* with all other estuarine bivalves was still relatively small, e.g. 0% for *M*. *gigas*, 1% for *M*. *edulis*, 15% for *M*. *arenaria* and 25% for *C*. *edule* (Fig 9, Table 4).

In December, there was no overlap between the isotopic niches of *C*. *edule* and *M*. *gigas*, whilst the isotopic niche of *M*. *edulis* partly overlaps with these two species (Fig 9, Table 4).

## Discussion

### Food availability

At the Balgzand tidal flats, the highest availabilities of the three primary food sources occurred at different times of the year. Marine phytoplankton had the highest availability in the spring, microphytobenthos peaked in summer, while freshwater phytoplankton had peak values in the autumn. In general, highest values of freshwater that enter this system are found in winter (up to 3000 m^3^ s^-1^) and lowest in summer with no water supply at all on some days [68]. In 2014, the supply of freshwater algae to the Wadden Sea was highest in autumn as a result of a combination of high freshwater discharge and the highest chlorophyll a concentration in Lake IJssel and lowest in April, mostly due to low levels of water discharge in that month.

Since several unicellular freshwater algal species can be cultured in media consisting up to 60% seawater for 96 hours [69], at least part of the freshwater phytoplankton should be able to survive in brackish waters for at least several days. Also the dead organic matter (detritus) can be a valuable food source for estuarine bivalves [40–43]. Because the local availability of these resources might also vary in time (e.g. tides, seasons) and space (e.g. as the result of sinking and resuspension), diet studies of estuarine bivalves by means of stable isotope analyses should include all potential resources (including freshwater SPOM) during the growing period. Sampling should then preferably be done close to the sediment within the full tidal window when bivalves have direct access to their food.

Ideally the isotopic signal of pure phytoplankton samples would be the best solution to exclude the effect of other particulate matter on the signal. However, this type of sampling is time consuming and might not be a realistic approach. An alternative would be the analysis of compound specific isotopic composition e.g. amino acids [70] or the inclusion of sulfur as an additional isotope to distinguish the influence of freshwater material [71, 72].

However, several of the averages of food sources in this study (all freshwater SPOM and microphytobenthos in December) were based upon single observations, leading to some uncertainties regarding the stable isotope values of the sources. Also within the mixing model correlations of up to 0.97 between two food sources were found. Such large correlations indicated that the model cannot discern between the two sources, which means that if one source is being consumed at the top of its probability range, then the other is likely to be at the bottom of its probability range, and vice versa [73].

### Seasonal variation in diet

During this study, two species (*Abra tenuis*, *Scrobicularia plana*) and one period (December) were undersampled and are, therefore, not further considered. In March, June and September 2014, the obligate suspension-feeding bivalves (*Cerastoderma edule*, *Magallana gigas*, *Mya arenaria* and *Mytilus edulis*) appeared to have predominantly utilized freshwater SPOM as their main food source. Only *Limecola balthica*, a bivalve species that can switch between deposit- and suspension-feeding [29, 74], appeared to have fed mainly on estuarine SPOM during spring. In June and September 2014, this Baltic tellin appears to have preferred microphytobenthos, most probably gathered by means of deposit-feeding.

It should be noted, however, that most correlations between food sources were high, indicating that the models could not discern very well between these resources [73]. These outcomes are, nevertheless, in concordance with previous findings that freshwater SPOM can be an important food source for estuarine obligate suspension-feeding bivalves [40–43] and that main food sources of estuarine facultative deposit-feeding bivalves can vary within the year [28].

The results of the mixing model are also highly depending on the trophic discrimination factor. Within this study we used values that were measured by Dubois [63], because these values were based upon a feeding experiment on two estuarine bivalve species also included in our research (*M*. *gigas* and *M*. *edulis*). These values differ from the average values reported in literature [72]; for δ^13^C we used 2 ‰ instead of 1 ‰ and for δ^15^N we used 3.78 ‰ instead of 2.2–3.4 ‰. Additional analyses (not shown here) with these lower trophic discrimination factors resulted in changes in the probabilities of the three food sources (up to 25–30%) in the diet of the bivalves. Using other values for trophic discrimination factors did, however, not change our main conclusion that all species feed on a mixture of different sources and that the freshwater SPOM is a relevant part of their diet.

### Ontogenic variation in diet

The significant correlation of the δ^13^C (*L*. *balthica*, *M*. *arenaria* and *M*. *edulis*) and the δ^15^N (*C*. *edule*, *M*. *gigas*, *M*. *arenaria* and *M*. *edulis*) signals with shell length suggests that the diet of these estuarine bivalves varies through life-cycle stages. This might be due to gradual shifts in their feeding behaviour in life, to different access to various food sources with size and/or to different timing in growing seasons with age. As was observed for edible cockles (*C*. *edule*) in the Gironde estuary [21], for example, seasonal variation in the availability of food resources combined with non-synchronous growing seasons of juvenile and adult bivalves (spring versus summer) might lead to shifts in main diets with size. In general, relationships between isotopic signals and size might also be explained by differences in growth rates, with juveniles generally growing faster than adults [75]. Additional to ontogenetic shifts in bivalves also shifts in the δ^15^N baseline are a possible explanation of the observed changes here. The small shift in δ^15^N between the March and December values and the slightly higher values during summer months, June and September could be due to a change in food web complexity and an increase in trophic level for most sampled organisms. However, it could also be an indication that the baseline δ^15^N values shifted a little from the winter to the summer situation. Based on the data presented here, this is all very speculative, but this question definitely deserves some further attention.

The negative relationship between the δ^13^C signal and shell length in combination with the ranges observed for these species indicates that, on average, the diet of *L*. *balthica* shifted from microphytobenthos to estuarine SPOM when growing from 5 to 25 mm shell length and that of *M*. *arenaria* from estuarine SPOM to freshwater SPOM when growing from 25 to 100 mm shell length. For Baltic tellins (*L*. *balthica*), this ontogenic change is in line with observations for the Scheldt estuary where small juveniles were feeding mostly on microphytobenthos, while larger sized bivalves tended to feed more and more on estuarine phytoplankton (Rossi et al. 2004). The shift in the soft-shelled clam *M*. *arenaria* might be due to thresholds for feeding behaviour as determined by low food concentrations [76]. If the threshold where this species stops feeding increases with its size, then this bivalve might prefer to feed more and more during food-rich waters caused by discharge outflows during its life time.

The positive relationship between the δ^13^C signal and shell length in combination with the ranges observed for the blue mussel *M*. *edulis* indicates that, on average, this species shifted from a relatively higher proportion of estuarine SPOM compared to freshwater SPOM in its diet when growing from 10 to 70 mm shell length. Mussels frequently change position and orientation during their life time (e.g. [77]). If small mussels settle, on average, at the top of a mussel bed and then gradually move down to lower locations as new mussels and oysters settle on top of them, they would get less and less access to the relatively light freshwater (containing freshwater SPOM) and more and more to the denser saline waters (containing marine SPOM).

Estuarine bivalves such as *C*. *edule* and *M*. *edulis* were found to ingest a wide range of zooplankton species, including bivalve larvae, selectively consuming smaller categories of zooplankton present [78]. If the threshold of edible zooplankton increases with shell size, then it is expected that the proportion of primary consumers increases during the life time of bivalves. Such an increase would then be reflected by a significant increase of the δ^15^N signal with shell length as was observed for *C*. *edule*, *M*. *gigas*, *M*. *arenaria* and *M*. *edulis*.

### Interspecific competition for food

Within the estuarine bivalve community, the interspecific niche overlap was highest in March. This seasonal variation in niche overlap was partly due to the variation in species-specific niche breadths. Such seasonal variation might indicate that the bivalves were relying on a wider array of resources in March than in June, but also that the bivalves were more food-limited in spring than in summer [75].

Although food limitation is not very likely because the relatively high niche breadth is mainly found within the obligate suspension-feeders during the phytoplankton spring bloom, this possible cause cannot be excluded. More probable, the bivalves took advantage of the high availability of the same food source at that time. Interspecific competition for food was more likely to occur during summer, when marine phytoplankton densities were low as the result of low nutrient concentrations and the supply of freshwater phytoplankton was low due to low inflows from Lake IJssel. During that time, microphytobenthos is probably the main potential food source for estuarine bivalves, which is in line with the findings by Christianen et al. [16]. For obligate suspension-feeding bivalves, their food source might only come available during high wind speeds when benthic algae are resuspended into the water column [25].

## Conclusions

Our results indicate that freshwater algae may contribute to the food supply of estuarine bivalves in the study area, and that diets of bivalves vary with season and shell length. Although not all parts of the Wadden Sea receive as much freshwater import as the western Wadden Sea, the estuaries of large rivers such as Ems, Elbe and Weser show comparable freshwater inflows [68]. Diet studies should evaluate all food sources utilized by temperate estuarine bivalves and sampling should include all possible resources and be in close proximity to the feeding space of these bivalves.

## Supporting information

S1 Fig Season-specific matrix plots of food sources (microphytobenthos, estuarine SPOM and freshwater SPOM) for estuarine bivalves in March, June and September 2014. (DOCX)

S1 Table Summary of the linear models (with fixed slope) of the relationship of δ^13^C with shell length (mm) and season (in comparison to June for *S. plana*, and to March for all other species) for the different bivalve species as sampled in March, June, September and December 2014 at the Balgzand tidal flats. (DOCX)

S2 Table Summary of the linear models (with fixed slope) of the relationship of δ^15^N with shell length (mm) and season (in comparison to June for *S. plana*, and to March for all other species) for the different bivalve species as sampled in March, June, September and December 2014 at the Balgzand tidal flats. (DOCX)

S3 Table Overview of stable isotope values. Seasonal mean, sd and number of samples (n) of stable isotope values of the different bivalve species collected at two stations on the Balgzand tidal flats in 2014, empty fields indicate that no samples have been collected, trophic guild based on Kamermans 1994 [18].
(DOCX)
