# Supplementary material for: Seasonal variation in the diet of estuarine bivalves
Source: PLoS One. 2019 Jun 17;14(6):e0217003. doi: 10.1371/journal.pone.0217003 (PMC6579449; doi:10.1371/journal.pone.0217003)
Supplement: S1 Fig — (DOCX) [file pone.0217003.s001.docx]

**S1 Fig.. Season-specific matrix plots of food sources (microphytobenthos, estuarine SPOM and freshwater SPOM) for estuarine bivalves in March, June and September 2014.**

| *Cerastoderma edule* |  |  |
| --- | --- | --- |
| 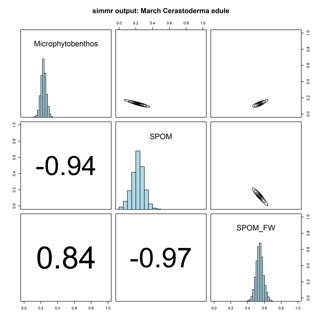 | 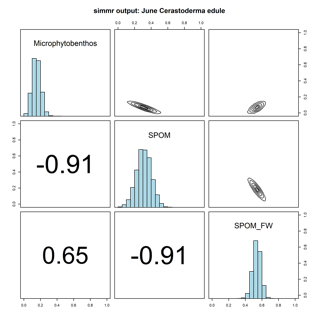 | 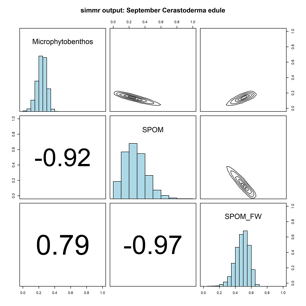 |
| *Magallana gigas* |  |  |
| 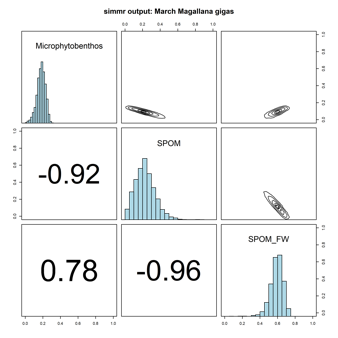 | 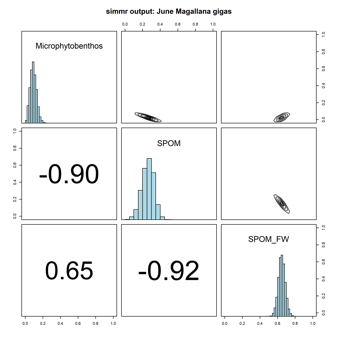 | 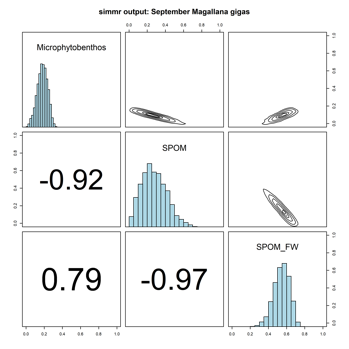 |
| *Limecola balthica* |  |  |
| 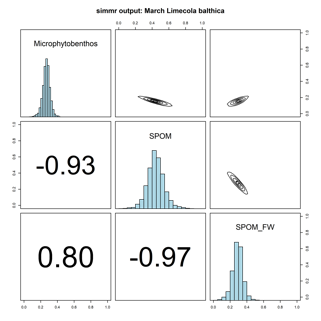 | 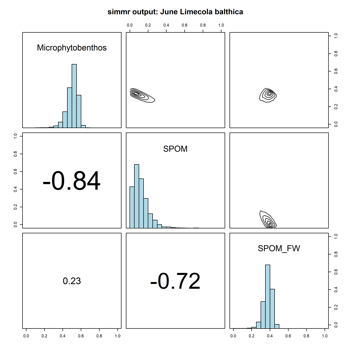 | 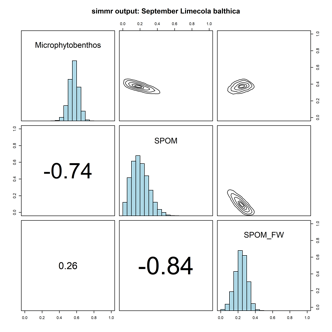 |

**S1 Fig. - Continued. Season-specific matrix plots of food sources (microphytobenthos, estuarine SPOM and freshwater SPOM) for estuarine bivalves in March, June and September 2014.**

| *Mya arenaria* |  |  |
| --- | --- | --- |
| 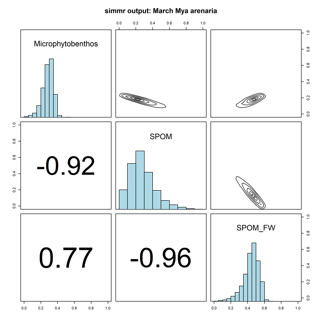 | 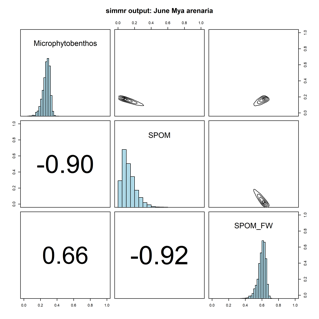 | 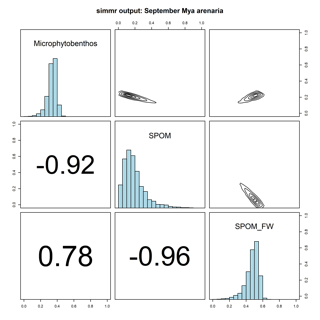 |
| *Mytilus edulis* |  |  |
| 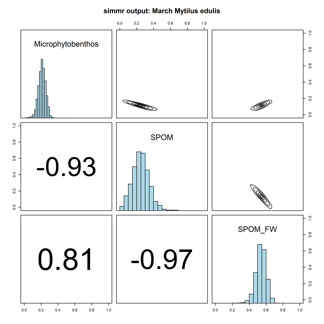 | 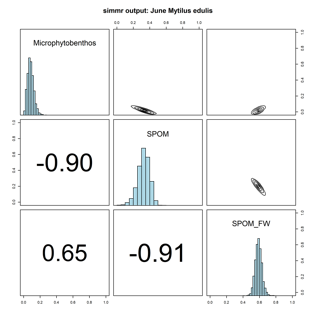 | 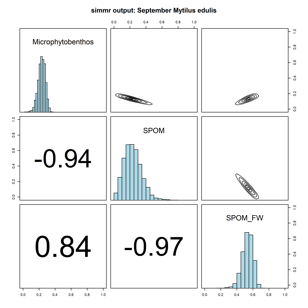 |
