# Supplementary material for: Seasonal variation in the diet of estuarine bivalves
Source: PLoS One. 2019 Jun 17;14(6):e0217003. doi: 10.1371/journal.pone.0217003 (PMC6579449; doi:10.1371/journal.pone.0217003)

**S2 Table. Summary of the linear models (with fixed slope) of the relationship of δ^15^N with shell length (mm) and season (in comparison to June for *S. plana*, and to March for all other species) for the different bivalve species as sampled in March, June, September and December 2014 at the Balgzand tidal flats.**
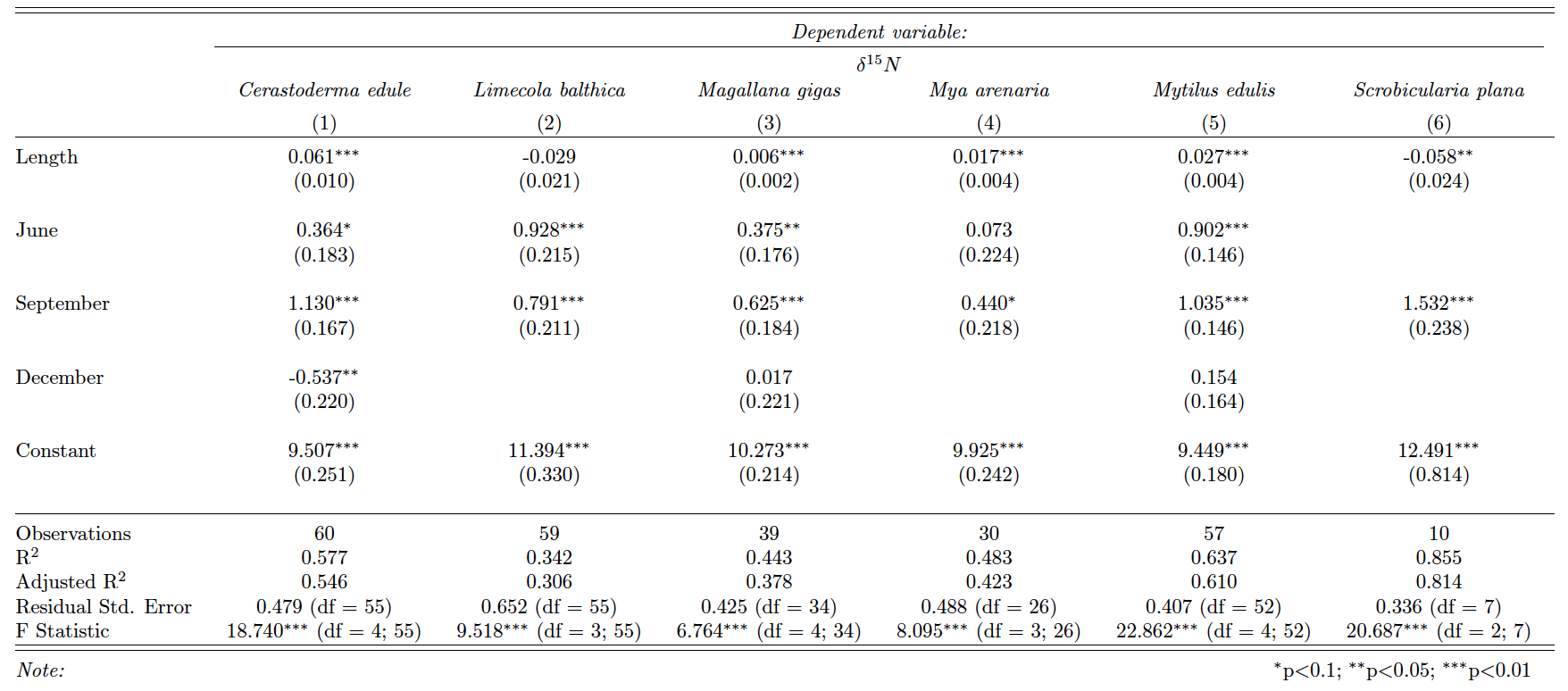

Supplement: S2 Table — (DOCX) [file pone.0217003.s003.docx]
