# Supplementary material for: Seasonal variation in the diet of estuarine bivalves
Source: PLoS One. 2019 Jun 17;14(6):e0217003. doi: 10.1371/journal.pone.0217003 (PMC6579449; doi:10.1371/journal.pone.0217003)
Supplement: S3 Table — Seasonal mean, sd and number of samples (n) of stable isotope values of the different bivalve species collected at two stations on the Balgzand tidal flats in 2014, empty fields indicate that no samples have been collected, trophic guild based on Kamermans 1994 [18]. (DOCX) [file pone.0217003.s004.docx]

**S3 Table. Overview of stable isotope values.** Seasonal mean, sd and number of samples (n) of stable isotope values of the different bivalve species collected at two stations on the Balgzand tidal flats in 2014, empty fields indicate that no samples have been collected, trophic guild based on Kamermans 1994 [18].

|  |  |  | March | | | | | June | | | | | September | | | | | December | | | | |
| --- | --- | --- | --- | --- | --- | --- | --- | --- | --- | --- | --- | --- | --- | --- | --- | --- | --- | --- | --- | --- | --- | --- |
| Species name | Trophic guild | Station | n | Mean δ^15^N | sd δ^15^N | Mean δ^13^C | sd δ^13^C | n | Mean δ^15^N | sd δ^15^N | Mean δ^13^C | sd δ^13^C | n | Mean δ^15^N | sd δ^15^N | Mean δ^13^C | sd δ13C | n | Mean δ^15^N | sd δ^15^N | Mean δ^13^C | sd δ^13^C |
| *Abra tenuis* | suspension feeder/ deposit feeder | 2 | 5 | 11.49 | 1.57 | -18.59 | 3.07 | 1 | 12.09 |  | -15.80 |  | 2 | 11.60 | 0.14 | -16.10 | 0.00 |  |  |  |  |  |
| *Cerastoderma edule* | suspension feeder | 1 | 15 | 11.21 | 0.48 | -20.33 | 0.61 | 4 | 11.88 | 0.25 | -19.53 | 0.17 | 5 | 11.78 | 0.73 | -19.39 | 0.62 | 1 | 9.50 |  | -19.00 |  |
| *Cerastoderma edule* | suspension feeder | 2 | 18 | 10.73 | 0.38 | -19.03 | 0.36 | 5 | 11.22 | 0.35 | -18.56 | 0.21 | 7 | 11.84 | 0.36 | -17.90 | 0.59 | 5 | 11.01 | 1.34 | -17.92 | 0.37 |
| *Limecola balthica* | suspension feeder/ deposit feeder | 1 | 31 | 10.82 | 0.59 | -18.03 | 1.04 | 6 | 12.51 | 0.31 | -16.79 | 0.74 | 5 | 11.98 | 0.30 | -16.42 | 0.08 |  |  |  |  |  |
| *Limecola balthica* | suspension feeder/ deposit feeder | 2 | 6 | 11.72 | 0.42 | -15.83 | 0.87 | 7 | 11.33 | 0.53 | -14.70 | 0.64 | 9 | 11.69 | 0.52 | -13.27 | 1.23 |  |  |  |  |  |
| *Magallana gigas* | suspension feeder | 1 | 13 | 10.83 | 0.40 | -20.42 | 0.83 | 4 | 11.38 | 0.36 | -20.05 | 0.47 | 1 | 12.10 |  | -19.90 |  | 1 | 9.70 |  | -20.20 |  |
| *Magallana gigas* | suspension feeder | 2 |  |  |  |  |  | 7 | 11.00 | 0.36 | -19.91 | 0.38 | 8 | 11.36 | 0.50 | -19.26 | 0.33 | 5 | 10.82 | 0.54 | -19.50 | 0.19 |
| *Mya arenaria* | suspension feeder | 1 | 7 | 10.54 | 0.51 | -18.89 | 0.70 | 5 | 11.39 | 0.32 | -18.70 | 0.31 | 4 | 11.58 | 0.51 | -18.43 | 0.36 |  |  |  |  |  |
| *Mya arenaria* | suspension feeder | 2 | 3 | 10.90 | 0.53 | -17.57 | 0.25 | 4 | 9.98 | 0.26 | -18.10 | 0.16 | 7 | 11.08 | 0.39 | -17.10 | 0.58 |  |  |  |  |  |
| *Mytilus edulis* | suspension feeder | 1 | 8 | 10.38 | 0.59 | -20.56 | 0.79 | 5 | 11.68 | 0.47 | -19.62 | 0.34 | 5 | 11.68 | 0.27 | -18.95 | 0.24 | 5 | 11.02 | 0.88 | -19.26 | 0.46 |
| *Mytilus edulis* | suspension feeder | 2 | 12 | 10.61 | 0.57 | -19.28 | 0.54 | 8 | 11.09 | 0.47 | -19.75 | 0.76 | 10 | 11.04 | 0.29 | -18.74 | 0.24 | 4 | 10.40 | 0.36 | -18.55 | 0.25 |
| *Scrobicularia plana* | suspension feeder/ deposit feeder | 2 | 2 | 11.55 | 0.64 | -16.30 | 0.28 | 6 | 10.53 | 0.52 | -17.13 | 0.44 | 4 | 11.82 | 0.17 | -15.38 | 0.65 |  |  |  |  |  |
